# Fungal identification in peanuts seeds through multispectral images: Technological advances to enhance sanitary quality

**DOI:** 10.3389/fpls.2023.1112916

**Published:** 2023-02-22

**Authors:** Julia Marconato Sudki, Gustavo Roberto Fonseca de Oliveira, André Dantas de Medeiros, Thiago Mastrangelo, Valter Arthur, Edvaldo Aparecido Amaral da Silva, Clíssia Barboza Mastrangelo

**Affiliations:** ^1^ Laboratory of Radiobiology and Environment, Center for Nuclear Energy in Agriculture, University of São Paulo (CENA/USP), Piracicaba, SP, Brazil; ^2^ Department of Crop Science, College of Agricultural Sciences, Faculdade de Ciências Agronômicas (FCA), São Paulo State University (UNESP), Botucati, Brazil; ^3^ Department of Agronomy, Federal University of Viçosa (UFV), Viçosa, Brazil

**Keywords:** *Arachis hypogaea* L., machine learning, *Aspergillus* spp., support vector machine, seed health

## Abstract

The sanitary quality of seed is essential in agriculture. This is because pathogenic fungi compromise seed physiological quality and prevent the formation of plants in the field, which causes losses to farmers. Multispectral images technologies coupled with machine learning algorithms can optimize the identification of healthy peanut seeds, greatly improving the sanitary quality. The objective was to verify whether multispectral images technologies and artificial intelligence tools are effective for discriminating pathogenic fungi in tropical peanut seeds. For this purpose, dry peanut seeds infected by fungi (*A. flavus*, *A. niger*, *Penicillium* sp., and *Rhizopus* sp.) were used to acquire images at different wavelengths (365 to 970 nm). Multispectral markers of peanut seed health quality were found. The incubation period of 216 h was the one that most contributed to discriminating healthy seeds from those containing fungi through multispectral images. Texture (Percent Run), color (CIELab L*) and reflectance (490 nm) were highly effective in discriminating the sanitary quality of peanut seeds. Machine learning algorithms (LDA, MLP, RF, and SVM) demonstrated high accuracy in autonomous detection of seed health status (90 to 100%). Thus, multispectral images coupled with machine learning algorithms are effective for screening peanut seeds with superior sanitary quality.

## Introduction

The peanut seeds (*Arachis hypogaea L*.) are of great food importance because, besides being a relevant source of vitamins, they are also rich in minerals, proteins and oil (Arya et al., 2016; Bessada et al., 2019). However, one factor that can limit the establishment of this and other important food crops is fungal contamination of the seeds, especially during post-harvest (Zhang et al., 2020; Wang et al., 2022). The infection of peanut seeds by pathogens such as *Penicillium* spp. (Aristil et al., 2020) and the *Aspergillus* spp. (Jayaprakash et al., 2019) compromises the sanitary quality and, consequently, seed performance (Wang et al., 2022). Traditional seed pathogen detection protocols (ISTA, 2020) are limited and subjective, requiring analytical expertise to obtain reliable results. This has been an obstacle to enhance the sanitary control of peanut seeds. Thus, investigating new methods capable of determining seed health is part of a technological strategy in agriculture. Considering that the peanut is produced and traded globally (USDA, 2022), we ask ourselves: how can we detect pathogenic fungi in seeds in a more efficient and less subjective way? The alternative proposed brings together technological strategies based on multispectral images and machine learning to optimize the analysis of the seed sanitary state.

Multispectral images have wide application in studies of seeds cultivated and forest species (Elmasry et al., 2019a; Barboza da Silva et al., 2021b). Their ability to assess multi-angle organic realities in the electromagnetic spectrum brings promising exploratory power for fungal detection (Boelt et al., 2018). The main reason for this is that the mycelia resulting from fungal colonization changes the surface and chemical composition of the seeds (Zhang et al., 2022). This event generates easily perceptible changes in the spectral range (França-Silva et al., 2020; Rego et al., 2020). In peanut seeds, information acquired at various wavelengths has made it possible to diagnose pathologies non-invasively (Qiao et al., 2017; Ziyaee et al., 2021). Some multispectral descriptors such as color (CIELab L*) and reflectance have high sensitivity for detecting the post-harvest decay status of peanut seeds (Fonseca de Oliveira et al., 2022). For instance, in a seed of an oleaginous species (*J. curcas*), textural parameters obtained from multispectral images make it possible to diagnose gray-level nuances associated with fungal contamination (Bianchini et al., 2021). Thus, the simultaneous analysis of color, reflectance and texture can increase the accuracy in detecting peanut seed pathogens.

The interaction of light with the physical and chemical characteristics of seeds can define their spectral identity. This information can be valuable for improving the characterization of the sanitary quality of the peanut seeds. In the case of the color and reflectance parameters, the brightness characteristics of the seed surface coat are indicative of aging of the embryonic tissues (Fonseca de Oliveira et al., 2022) or infection by pathogenic organisms (Boelt et al., 2018). The seed reflectance is altered in the presence of the pathogen mycelia, which makes it possible to distinguish fungal species with high accuracy (França-Silva et al., 2020). Also, the reflectance property depends on parameters such as the texture and chemical composition of the fungal colonies and the seeds (Barboza da Silva et al., 2021a). The development of the fungus depends on the consumption of reserves in the embryo (Zhang et al., 2022), which physically modifies the integument with the formed mycelia. These chemical and structural changes in the seed generate variations in reflectance (Ziyaee et al., 2021) and in textural parameters such as smoothness, roughness and regularity that can be detected autonomously (Rego et al., 2020). In addition, these descriptors can reveal significant differences in pathogens that appear similar to human visual inspection (Elmasry et al., 2019a). Therefore, by exploring multispectral descriptor technologies, an opportunity is created to improve the technical rigor of the sanitary control in the peanut seeds.

These multispectral image technologies coupled with artificial intelligence algorithms can contribute to decision making ensuring high standards of peanut seed health. Machine learning is a digital analytical resource for predicting patterns based on numerical data (Medeiros et al., 2020a; Medeiros et al., 2020b). It is a branch of artificial intelligence with wide application, including studies with seeds (Barboza da Silva et al., 2021b; Batista et al., 2022; Fonseca de Oliveira et al., 2022). The simplicity and high efficiency of these features can reduce analysis time (Elmasry et al., 2019a). Autonomous decision making from the large volume of data generated by multispectral images can streamline peanut seed quality management. This proposal has been documented for seeds of other species (França-Silva et al., 2020; Rego et al., 2020; Barboza da Silva et al., 2021a). The research hypothesis is that multispectral image technology coupled with machine learning algorithms can optimize the identification of the sanitary state of peanut seeds. Therefore, the aim was to verify whether this technique could effectively discriminate fungi that occur most commonly in the peanut seeds.

## Materials and methods

### Plant material

The peanut seeds (IAC 503 cultivar) used in this study were kindly provided by Dr. Ademir Hilário Amaral, Seed Quality Manager at the Sementes Esperança Company (https://www.sementesesperanca.com.br/) in Jaboticabal, São Paulo, Brazil.

### Trial design

Here, the idea was to investigate technological tools with the potential to access the sanitary quality of peanut seeds. For this, commercial peanut seeds inoculated with different types of pathogenic fungi were used. Initially, the fungi were analyzed in a traditional test (blotter method). Subsequently, images were obtained at different wavelengths of these seeds according to the incubation time of the fungi. To validate the information, verified through the multispectral images of the seeds, machine-learning algorithms were used. In summary, we demonstrated the possibility of detecting pathogenic fungi in peanut seeds by imaging parameters. Many of them, not detected by human vision in traditional analyses. Therefore, here we present a new method for peanut seed health analysis with potential application in monitoring the sanitary quality of commercial seed lots.

### Fungal isolation, inoculation, and incubation

The isolates of *Aspergillus flavus*, *Aspergillus niger*, *Penicillium* sp. and *Rhizopus* sp. were obtained from 500 peanut seeds using the blotter method (500 seeds for each fungal isolation). Peanut seeds were distributed in a 9 cm plastic Petri dish (five seeds per Petri dish) containing three layers of sterilized blotting paper moistened with 6 mL sterile distilled water and kept at 20°C with a 12 h photoperiod of white fluorescent light for 7 days. The seeds were examined individually, and the fungi were identified based on morphological characteristics using a stereomicroscope. Next, the fungi were picked with a sterile needle and transferred to sterile 9 cm Petri dishes containing potato dextrose agar (PDA). The Petri dishes were kept in a growth chamber at 20°C with a 12 h photoperiod of white fluorescent light for 10 days.

After this process, disinfected seeds, asepsis with 1% sodium hypochlorite for 3 min (Mahajan et al., 2018; Doncato and Costa, 2019), were put in direct contact with the fungal colonies and kept in a growth chamber under the same conditions described above for 24 h. The disinfection of the seeds before inoculation of the fungus allowed to generate information without the possible influence of other microorganisms present in the seed or associated with it through the air or soil during its production in the field. A group of disinfected seeds was separated to form a control (healthy seeds). The peanut seeds were dried on three layers of paper towels at room temperature for 24 h. Afterwards, the infected seeds were incubated under the same conditions previously described for 24, 48, 72, 96, 120, 144, 168, 192, 216 and 240 h.

### Procedures for extracting multispectral data

After incubation, 500 seeds from each incubation period (24, 48, 72, 96, 120, 144, 168, 192, 216 and 240 h) and from the control treatment (healthy seeds) were placed on a transparent acetate sheet (5.0 cm x 8.5 cm) in the same position using double-sided adhesive tape. Before image acquisition, the light setting was adjusted to optimize the strobe time of each illumination type and improve signal-to-noise ratio such that the captured images could be directly comparable. Light setup was calibrated using a representative sample and saved for all subsequent images. The multispectral images (spatial dimension of 2192 x 2192 pixels; 40 μm/pixel) were captured using a VideometerLab4 device (Videometer A/S, Herlev, Denmark, software version 5.4.6). The samples were then illuminated with 19 monochromatic light-emitting diodes (LEDs: 365, 405, 430, 450, 470, 490, 515, 540, 570, 590, 630, 645, 660, 690, 780, 850, 880, 940 and 970 nm). The ambient light was switched off during the image acquisition to remove false light coming into the image. RGB (Red-Green-Blue) images were also acquired using the same sensor.

A segmentation technique based on thresholding was used to remove the background and isolate the region of interest – ROI (peanut seed + fungus). A processing algorithm based on normalized canonical discrimination analysis (nCDA) was applied to the images to highlight the intensity of the reflectance signals pixel by pixel (Bianchini et al., 2021). This algorithm uses the trimmed mean of pixel values, eliminating the influence of outliers (the lowest 10% and the highest 10%) (Barboza da Silva et al., 2021a), and then, it transforms grayscale images into score images with red-green-blue color codes. Different texture, color and reflectance descriptors were extracted with a Binary Large Object (BLOB) tool in the VideometerLab software. This tool serves to detect the object under analysis (i.e. seeds), automatically segment the image by making the pixels in the background null, and extract the data individually for each object.

Texture descriptors were extracted from the images to characterize the structural arrangement of the fungal mycelia, which was calculated using mathematical models proposed by Galloway (1975); Chu et al. (1990) and Albregtsen and Nielsen (2000). These models are based on a two-dimensional matrix called run-length matrix *p*(*i*, *j*), containing the number of runs of different gray lengths (*j*) and levels (*i*), arranged according to the gray lengths and values. Consecutive pixels of the same gray level in a given direction constitute a run, and the run length is the number of pixels in the run (Galloway, 1975). In general, the run-length matrix aims to calculate the number of consecutive pixels in a given direction that has the same gray-level intensity. For instance, a coarse texture will be dominated by relatively long runs, whereas a fine texture will predominantly have shorter runs (Nailon, 2010; Agwu and Ohagwu, 2016).

The run-length emphasis describes a number of consecutive pixels with the same gray-level value. It could be suitably termed long or short-run emphasis depending on the number of consecutive pixels in the chosen direction with the same grey-level value (Tang, 1998; Agwu and Ohagwu, 2016). The run-length and gray-level non-uniformity describe the pixel disorderliness and pixel grey-level runs. The fraction of the image in runs simply refers to run percentages, i.e., the ratio of the total number of runs in the image to the total number of pixels in the image expressed as a percentage (Tang, 1998; Agwu and Ohagwu, 2016).

Seven texture descriptors were extracted from the images: (i) short run emphasis (SRE), which measures the distribution of short runs, where the higher the SRE the finer the texture; (ii) long run emphasis (LRE), which measures the opposite, i.e., the higher the LRE the rougher the texture; (iii) gray-level nonuniformity (GLN), which assesses the distribution of runs between gray levels; (iv) run length nonuniformity (RLN), which measures how close the run lengths are in the image (the closer the run lengths are, the lower the RLN); (v) run percentage (RP), which measures the ratio between the number of runs and the maximum number of runs in a specific direction (linear structures will have the lowest RP values); (vi) low gray-level run emphasis (LGRE), which measures the distribution of the lowest gray level values ​​in an image; (vii) high gray-level run emphasis (HGRE), which measures the distribution of the highest gray level values. The texture descriptors were calculated using the following formulas:


$$\mathrm{1)}\;SRE=\frac{1}{n_{r}}\sum_{i=1}^{M}\sum_{j=1}^{N}\frac{p(i,j)}{j^{2}}$$

$$\mathrm{2)}\;LRE=\frac{1}{n_{r}}\sum_{i=1}^{M}\sum_{j=1}^{N}j^{2}p(i,j)$$

$$\mathrm{3)}\;GLN=\frac{1}{n_{r}}\sum_{i=1}^{M}{\left(\sum_{j=1}^{N}p(i,j)\right)}^{2}$$

$$\mathrm{4)}\;RLN=\frac{1}{n_{r}}\sum_{j=1}^{N}{\left(\sum_{i=1}^{M}p(i,j)\right)}^{2}$$

$$\mathrm{5)}\;RP=n_{r}/n_{p}$$

$$\mathrm{6)}\;LGRE=\frac{1}{n_{r}}\sum_{i=1}^{M}{\sum_{j=1}^{N}p(i,j)/i}^{2}$$

$$\mathrm{7)}\;HGRE=\frac{1}{n_{r}}\sum_{i=1}^{M}\sum_{j=1}^{N}i^{2}p(i,j)$$

Where, *p*(*i*, *j*) represents the number of runs with pixels of a gray level intensity equal to *i* and run length equal to *j* along a specific direction; *M* is the number of gray levels; *N* is the maximum run length in the image; *nr* is the total number of runs and *np* is the number of pixels in the image.

To obtain accurate information regarding any color differences between the fungal mycelia, an RGB color model was converted to the CIE L* a* b* color space, which comprises all colors perceptible to the human eye under controlled conditions. The CIE L* a* b* color space is represented by the perceived brightness L* and the chromaticity coordinates a* and b*. The intensity-hue-saturation (IHS) fusion technique was also applied to combine data from all spectral bandwidths. The CIE L* a* b* coordinates were calculated from the *XYZ* tristimulus values using the following formulas:


$$\mathrm{1)}\;L*=116{\left(\frac{Y}{Yn}\right)}^{\frac{1}{3}}-16$$

$$\mathrm{2)}\;a*=500\left[{\left(\frac{X}{X_{n}}\right)}^{1/3}-{\left(\frac{Y}{Yn}\right)}^{1/3}\right]$$

$$\mathrm{3)}\;b*=200\left[{\left(\frac{Y}{Y_{n}}\right)}^{1/3}-{\left(\frac{Z}{Zn}\right)}^{1/3}\right]$$

Where, L* represents the brightness ranging from 0.0 (black) to 100.0 (white); the dimensions a* and b* are correlated to the colors red-green and yellow-blue, respectively ranging from -120.0 (-a* = green; -b* = blue) to 120.0 (a* = red; b* = yellow); *X*, *Y* and *Z* are original tristimulus values, and *Xn*, *Yn* and *Zn* are tristimulus values of the reference white point.

Finally, to verify the influence of anthocyanin pigments on fungal spectral patterns (Fonseca de Oliveira et al., 2022), we also captured multispectral images using a SeedReporterTM instrument (PhenoVation B.V., Wageningen, Netherlands).

### Principal component analysis

To classify the peanut seeds contaminated with different fungi, a multivariate analysis technique based on PCA was used to identify patterns, correlations and contributions of the texture, color, and reflectance descriptors for discrimination of the different fungal classes. A correlation matrix was obtained through an original matrix *n* x *p*, where *n* was the number of seeds and *p* the number of variables, which was decomposed into eigenvalues and eigenvectors with the eigenvectors forming latent variables (called Component 1, Component 2, …, and Component *p*) composed of the linear combination of variables and their respective weights. The eigenvalues were used to verify the total percentage of variance explained by each of the principal components (Jollife and Cadima, 2016). The PCA allowed the identification within each parameter (texture, color, and reflectance) of the most accurate metric to distinguish the treatments (healthy seeds and 4 fungus classes). From the variables with the greatest contribution detected in the PCA, was identified which incubation time interval contributed the most to capture the greatest variability among the fungal classes.

### Machine learning - creation models for classifying fungal-infected peanut seeds

Four algorithms were used for data modeling: linear discriminant analysis (LDA), multi-layer perceptron neural network (MLP), support vector machine (SVM) and random forest (RF). The models were developed based on five classes (healthy seeds, seeds contaminated with *A. flavus*, *A. niger*, *Penicillium* sp. and *Rhizopus* sp.), using data from 500 seeds in total (for each treatment: 2500 analyzed data), in which 75% of the data (n = 1875) were used for training and cross-validation. (K-fold = 5) and 25% (n = 625) for independent validation of the models. In the modeling analysis, all variables were used (texture: GLN, HGRE, LRE, SRE, LGRE, RLN and RP; color: CIELab b*, CIELab a*, CIELab L*; reflectance (395 to 970 nm). The metrics used to measure the performance of the models were accuracy, Cohen’s Kappa coefficient, precision, recall, F1, and confusion matrix (documentation can be found at https://scikit-learn.org/stable/modules/model_evaluation.html). Python 3.6.0 was used to develop machine learning models.

### Analysis of variance

The data obtained from the multispectral images of 500 seeds provided by the variables with the greatest contribution to discriminating the sanitary quality of peanut seeds were submitted to ANOVA (each seed as a repetition; *n*=500) after checking the assumptions of normality and homoscedasticity using the Shapiro-Wilk and Bartlett tests. The averages were compared using the Tukey test (*P*< 0.05). This statistical analyses were performed using the R 4.0.3 software program (completely randomized design) (R Core Team, 2022).

## Results

### Identification of relevant spectral features based on texture, color, and reflectance data

**Figure 1:**
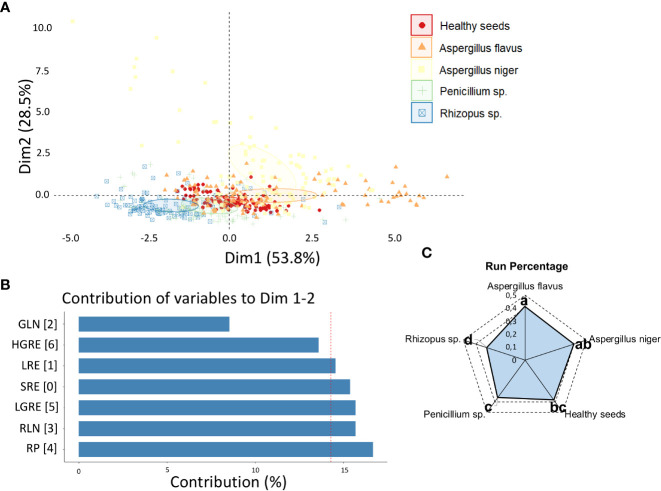
**(A)** Principal component analysis (PCA) of healthy peanut seeds and seeds infected by *Aspergillus flavus*, *Aspergillus niger*, *Penicillium* sp. and *Rhizopus* sp. based on texture descriptors; **(B)** contribution of texture descriptors for discrimination of different classes of fungi; **(C)** means of the texture descriptor that most contributed to the variability between fungal classes, i.e., RP. SRE, Short Run Emphasis; LRE, Long Run Emphasis; GLN, Gray-Level Nonuniformity; RLN, Run Length Nonuniformity; RP, Run Percentage; LGRE, Low Gray-Level Run Emphasis; HGRE, High Gray-Level Run Emphasis.

The two principal components (DIM 1 + DIM 2) explained 82.3% of the variation between classes based on texture descriptors (**Figure 1A**). The variability between the different classes was mainly influenced by the RP descriptor (**Figure 1B**), with higher and lower values for *Aspergillus flavus* and *Rhizophus* sp. groups, respectively (**Figure 1C**).

**Figure 2:**
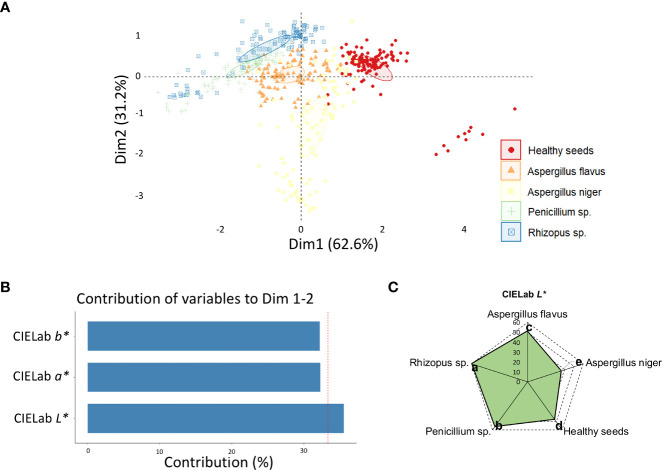
**(A)** Principal component analysis (PCA) of healthy peanut seeds and seeds infected by *Aspergillus flavus*, *Aspergillus niger*, *Penicillium* sp. and *Rhizopus* sp. based on color descriptors; **(B)** contribution of color descriptors for discrimination of different classes of fungi; **(C)** means of the color descriptor that most contributed to the variability between fungal classes, i.e., CIELab L*.

The Dim 1 + Dim 2 explained 93.8% of the data variability based on color descriptors (**Figure 2A**) with better separation of the classes compared to texture descriptors (**Figure 1A**). The variable that most contributed to the variability of the data was CIELab L* (**Figure 2B**), with a clear separation of the five classes, particularly *Rhizopus* sp. from *Aspergillus niger* (**Figure 2C**).

**Figure 3:**
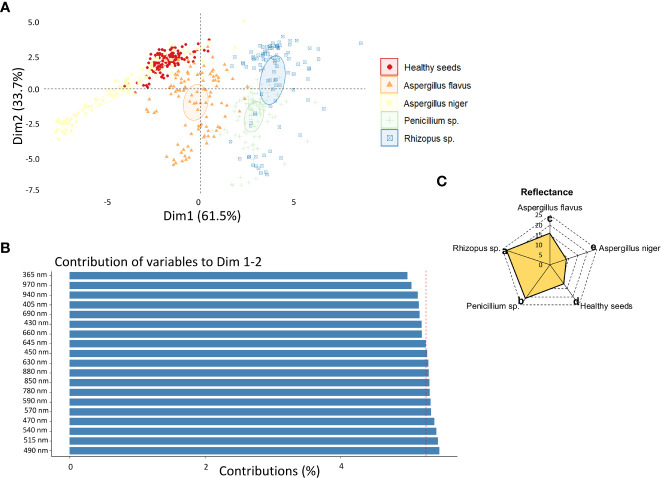
**(A)** Principal component analysis (PCA) of healthy peanut seeds and seeds infected by *Aspergillus flavus*, *Aspergillus niger*, *Penicillium* sp. and *Rhizopus* sp. based on reflectance descriptors; **(B)** contribution of reflectance descriptors for discrimination of different classes of fungi; **(C)** means of the reflectance descriptor that most contributed to the variability between fungal classes, i.e., 490 nm.

The two principal components explained a total of 95.2% (Dim 1 + Dim 2) of the variance between reflectance data (**Figure 3A**), in which the 490 nm band had the greatest contribution to the sorting of the classes (**Figure 3B**). At 490 nm, *Rhizopus* sp. showed the highest reflectance values, followed by *Penicillium* sp. and *Aspergillus flavus*, while the lowest values were obtained in seeds infected by *Aspergillus niger* (**Figure 3C**).

### Influence of the fungal development stage on inspection of peanut seed health

**Figure 4:**
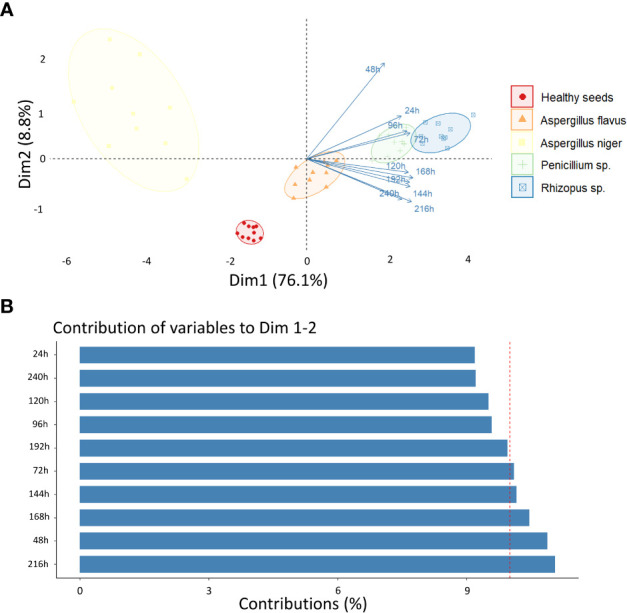
**(A)** Principal component analysis (PCA) of healthy peanut seeds and seeds infected by *Aspergillus flavus*, *Aspergillus niger*, *Penicillium* sp. and *Rhizopus* sp. after 24, 48, 72, 96, 120, 144, 168, 192, 216 e 240 h of incubation; **(B)** Contribution of incubation periods for discrimination of different classes of fungi.

**Figure 5:**
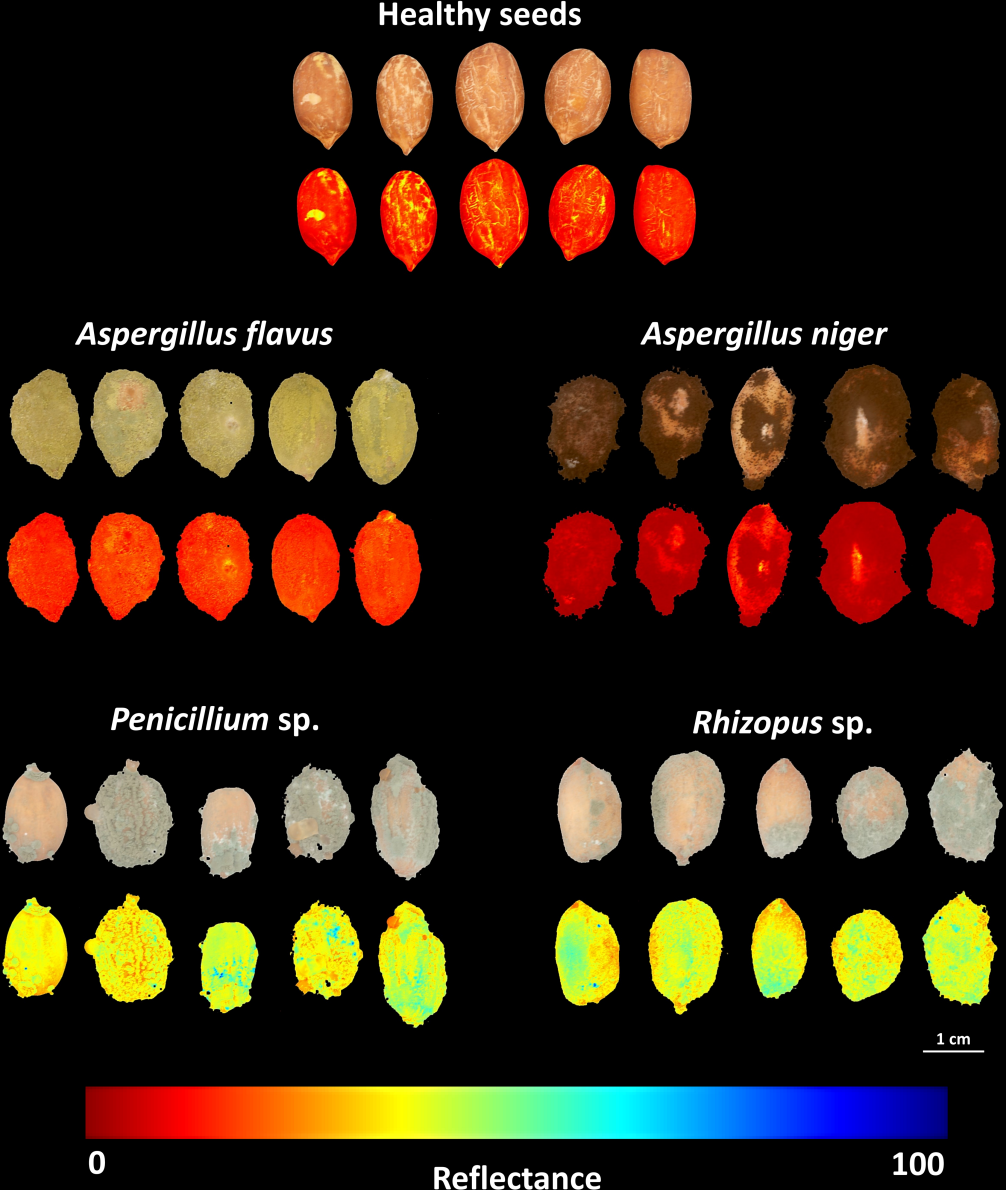
RGB (*Red-Green-Blue*) images and reflectance images captured at 490 nm from healthy peanut seeds and seeds infected by *Aspergillus flavus*, *Aspergillus niger*, *Penicillium* sp. and *Rhizopus* sp. after 216 h of incubation. The reflectance imagens were transformed by a normalized canonical discrimination (nCDA) algorithm; each pixel in the reflectance image contains a unique reflectance value dependent on the color, texture, and chemical composition of the fungal mycelia.

**Figure 6:**
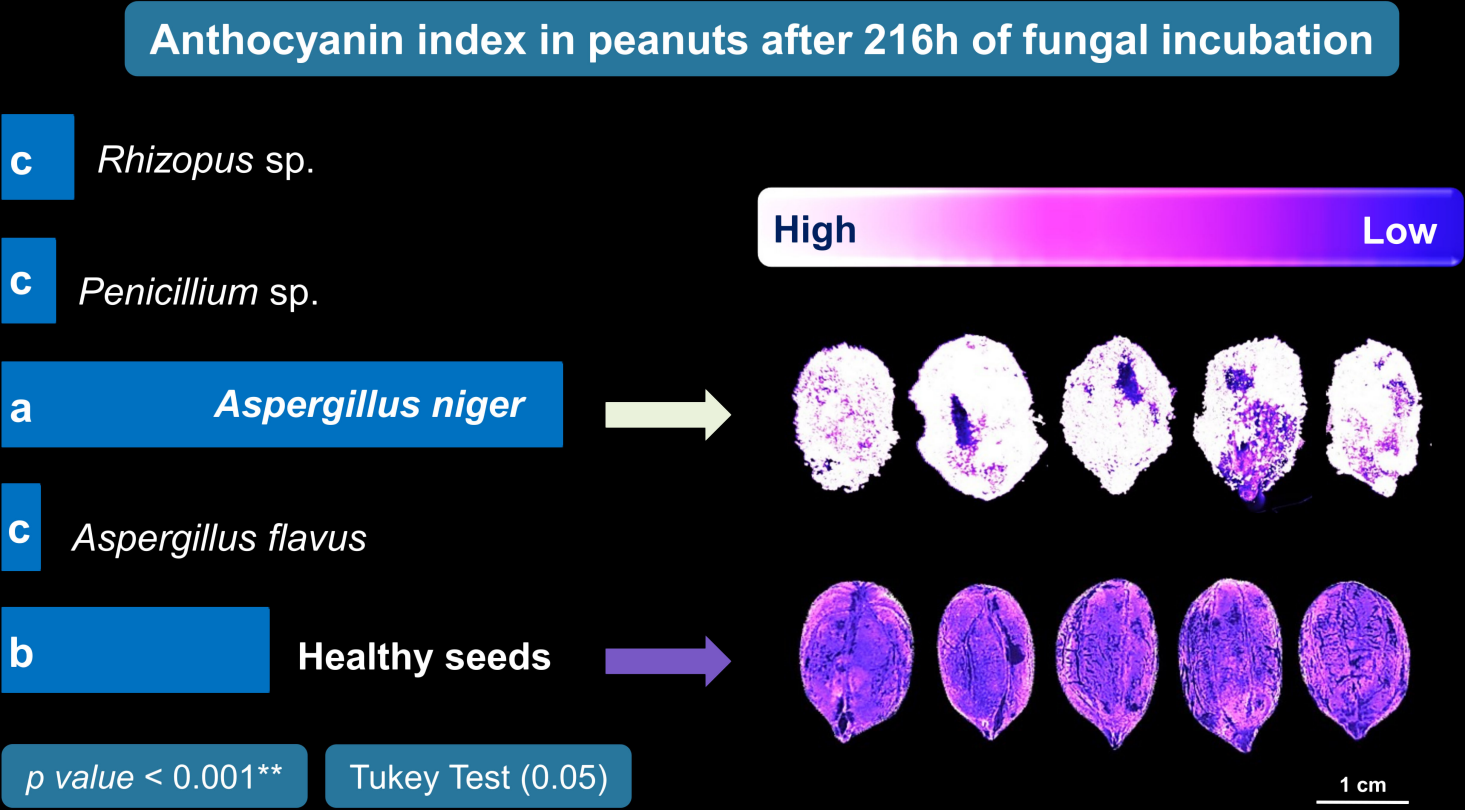
Anthocyanin index in peanut seeds infected by *Aspergillus flavus*, *Aspergillus niger*, *Penicillium* sp. and *Rhizopus* sp. after 216 h of incubation.

In order to identify the incubation period that most contributed to discriminate the different classes, an exploratory analysis of the data obtained for each incubation time was carried out based on PCA (**Figure 4**). This analysis was carried out considering the main discriminating parameters of the sanitary quality of peanut seeds, that is, RP (texture), CIELab *L* (color) and 490 nm (reflectance). The two principal components explained 84.9% (Dim 1 + Dim 2) of the variability between the data with a clear separation of the groups, although there was a high correlation between *Penicillium* sp. and *Rhizopus* sp. classes (**Figure 4A**). Spectral pattern differences were influenced mainly in the incubation period of 216 h (**Figure 4B**). For instance, in this period, the images captured at 490 nm and transformed by the nCDA algorithm proved the strong relationship of the spectral patterns of *Penicillium* sp. and *Rhizopus* sp. (**Figure 5**). In addition, the *Aspergillus niger*-infected seeds showed enhanced anthocyanin index compared to the healthy seeds (**Figure 6**).

### Machine learning models based on different multispectral approaches

The models created based on texture descriptors reached higher hit rates in the classification of *Aspergillus flavus, Aspergillus niger*, and *Rhizopus* sp. (**Supplementary Figure 1**), with better performance for the MLP algorithm (**Supplementary Table 1**). However, since there were high incidences of false-positives and false-negatives in the classification of *Penicillium* sp. for all algorithms. The models did not achieve satisfactory performance metrics, reaching a maximum accuracy of 67% (**Supplementary Table 1**).

Models based on color descriptors showed higher hit rates with the SVM algorithm, particularly in the recognition of *Aspergillus niger* (**Supplementary Figure 2**), with an accuracy of 82% (**Supplementary Table 1**). Using reflectance descriptors, all machine learning models reached satisfactory hit rates (**Supplementary Figure 3**), except for *Penicillium* sp. based on RF (0.67) and the SVM (0.07) algorithms. However, the models had perfect recognition of healthy seeds (LDA) and seeds contaminated with *Aspergillus niger* (LDA and SVM) and *Rhizopus* sp. (LDA, MLP and SVM), achieving accuracies ranging from 80% to 98% (**Supplementary Table 1**).

**Figure 7:**
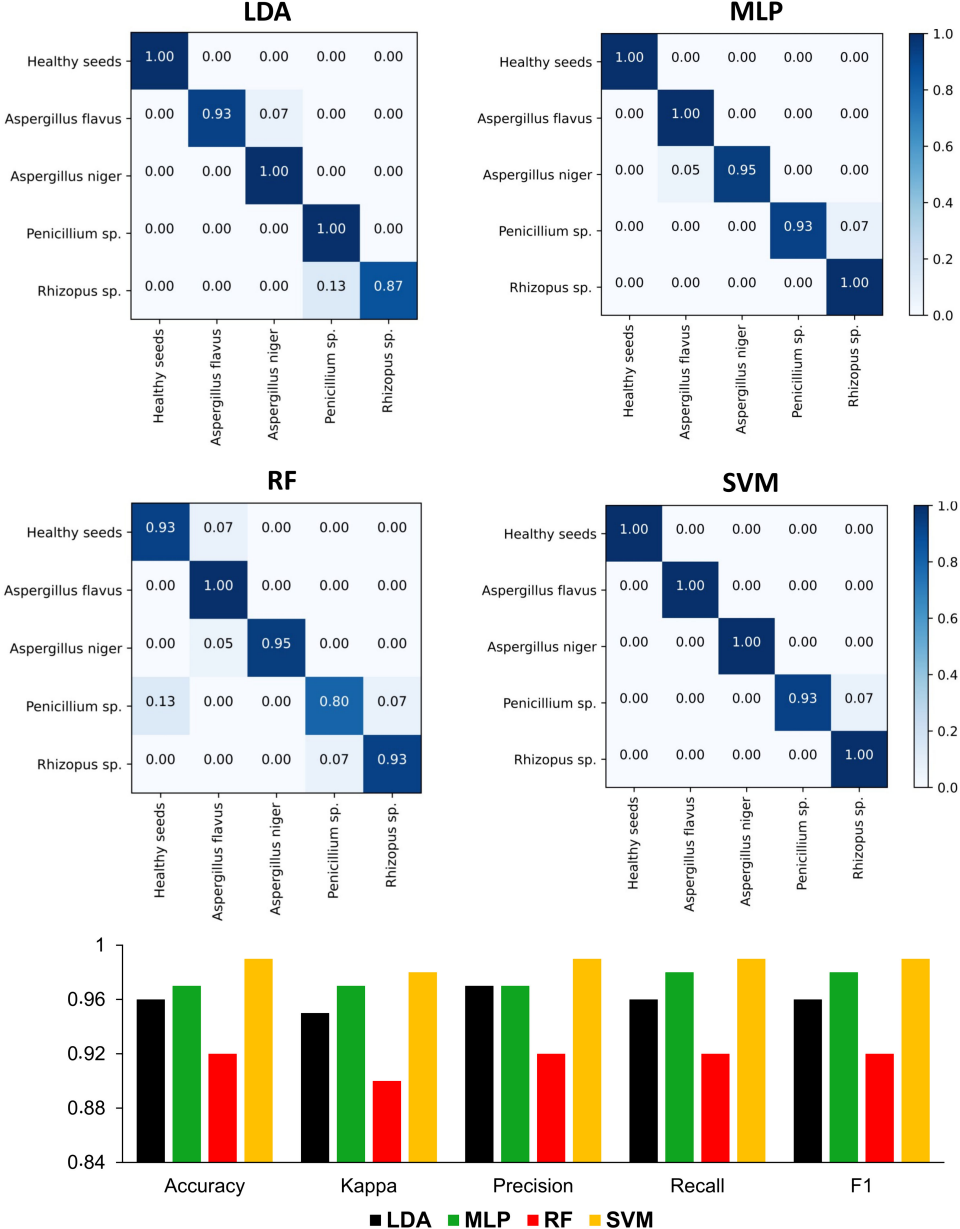
Confusion matrices and performance metrics of machine learning models based on linear discriminant analysis – LDA, multi-layer perceptron neural network – MLP, random forest – RF and support vector machine – SVM using a test dataset for classifying healthy peanut seeds and seeds contaminated with different groups of fungi using the combination of texture, color, and reflectance descriptors of multispectral images.

When machine learning models were developed using all parameters combined (i.e., texture, color, and reflectance) (**Figure 7**), there was an improvement in the performance, particularly for RF and SVM algorithms, which had showed high false-positive and false-negative rates in the classification of *Penicillium* sp. using only reflectance data (**Supplementary Figure 3**). Combining texture, color and reflectance descriptors, the rate of correctly classified *Penicillium* sp. increased from 0.67 to 0.80 for RF models (92% accuracy; 90% Kappa; 92% precision; 92% recall; 92% F1), and from 0.07 to 0.93 for SVM models (99% accuracy; 98% Kappa; 99% precision; 99% recall; 99% F1) (**Figure 7**). In general, the best performance was verified for the SVM algorithm, with a correct classification of 1.00 for healthy seeds, and seeds contaminated with *Aspergillus flavus*, *Aspergillus niger* and *Rhizopus* sp. (**Figure 7**).

## Discussion

We bring here technological approaches applied to accesses the sanitary quality of peanut seeds. Using multispectral images, we identified peanut seeds containing fungal infection with high accuracy. The insights brought by our results go beyond the limitations of traditional methods for seed health analysis (ISTA, 2020), which is known to be subjective. By using these imaging technologies together with machine learning capabilities, we propose an autonomous method of fungi detection in peanut seeds. Thus, new methods to mitigate fungal contamination in peanut seeds were investigated here.

The texture of the peanut seeds analyzed proved to be a highly effective spectral descriptor of their sanitary state. Fungal organisms colonizing the seeds physically alter the tegument through the mycelia formed and cause changes to its surface (França-Silva et al., 2020; Rego et al., 2020). This event is remarkable from a spectral point of view, because it affects the interaction of light with infected tissue and generates distinct levels of gray that can be identified digitally (Bianchini et al., 2021; Barboza da Silva et al., 2021a). We found here, that these spectral changes can characterize healthy or diseased seeds (**Figure 1A** and **Figure 1B**), as documented by other studies (Qiao et al., 2017; Ziyaee et al., 2021). In this work, the texture parameter Run Percentage (RP) was able to accurately discriminate fungal infection in the studied seeds (**Figure 1C**). For instance, in the present work, the fungus *Rhizopus* sp. showed the lowest RP value, indicating that this class of fungi has more linear structures. It also generated data that assists human vision in the identification of pathogens of global sanitary relevance. Overall, texture is a promising marker of peanut seed health. However, additional color parameters showed a similar technological application. This means that complementary results were generated with the potential to make the seed health analysis even more assertive.

We found that peanut seeds had consistent color distinctions (**Figure 2A**). This behavior, especially expressed by the CIELab L*, accurately translated gradual variations of brightness between infected and healthy tissue (**Figure 2B**). Each pathogen presented a luminosity identity characteristic of the genus and species studied (**Figure 2C**), which is hardly perceptible to the naked eye. Computer vision has extended the human sensory capacity to detect color differences, which is useful for assessing the appearance of in organic tissues (Altmann et al., 2022). This is because parameters such as luminosity (**Figure 2**) are numerically interpreted from multispectral images and express organic variations in a less subjective way (Brühl and Unbehend, 2021). In the sanitary field, this is interesting because it can facilitate the identification of pathogenic organisms in seeds (Rego et al., 2020) and mitigate their occurrence in post-harvest. This can avoid potential negative effects on plant establishment in the field that reduce yields (Ebone et al., 2020). By imaging across broad spectra, we can ensure the selection of peanut seeds with superior sanitary quality through non-invasive color analysis. Also, for other cultivated species, spectral imaging can discriminate physical and physiological characteristics that impact seed quality and agricultural performance (Hu et al., 2020; Bello and Bradford, 2021). Therefore, the color analysis of peanut seeds, in addition to other spectral characteristics, comprises an opportunity to improve the visual interpretation of contaminated seeds.

The third spectral feature we highlight for detecting pathogens in peanut seeds is reflectance (**Figure 3A**). This property has the potential to translate light reflected by infected seeds into information that differentiates fungal species (França-Silva et al., 2020; Rego et al., 2020). In the present study, contaminated seeds were accurately distinguished from healthy seeds by their superior reflectance in the 490 nm range (**Figure 3B**). These results may be associated with three aspects: i) the mycelia formed by *Rhizopus* sp., *Penicillium* sp. and *A. flavus* enhanced the brightness of the seeds (**Figure 2C**). This behavior contributed to the increase in reflectance (Fonseca de Oliveira et al., 2022); ii) the formation of mycelia and toxicogenic compounds alters the chemical composition of infected seeds (Zhang et al., 2022), which can reduce light absorption and favor reflection enhancement in the spectral range (**Figure 3C**); iii) fungal colonization intensifies the production of anthocyanins as a plant response to tissue deterioration (Liu et al., 2018; Fonseca de Oliveira et al., 2022). This pigment, is also produced by fungi (Bu et al., 2020; Sicilia et al., 2021) and its accumulation in the contaminated seeds (**Figure 6**) may have contributed to the lower reflectance found (**Figure 3C**). In peanut studies, reflectance has been exploited in infrared bands to detect fungi (Qiao et al., 2017; Xing et al., 2019; Conceição et al., 2021). Here, the fungi were observed in the visible spectrum (490 nm) which expands the chances of detecting organisms of seed sanitary importance (**Figure 5**). We highlight that from an optimum incubation time (216 h), each pathogen studied showed a predictable spectral nature when reflectance was analyzed together with texture and color parameters (**Figures 4A, B**). This indicates that our results add to the evidence in the literature and reinforce the potential of these parameters as markers of peanut seed health.

Given the technological potential discussed, how can we ensure speed and efficiency in the interpretation of peanut seed health in face of the abundant volume of information obtained through multispectral images? By exploring the potential of machine learning algorithms, we found they have excellent accuracy for this task (**Figure 7**). This means that human subjectivity can be mitigated in sanitary analysis while increasing assertiveness in recognizing healthy seeds. Human visual decision based on color, texture, and reflectance (i.e., 490 nm) is hardly sensitive enough to capture spectral nuances relevant for a more assertive pathological analysis (from 90 to 100% accuracy). The universe of intelligent machines brings possibilities for continuous improvements in the seed industry (Elmasry et al., 2019b; Medeiros et al., 2020b). This includes the identification of fungi (França-Silva et al., 2020; Rego et al., 2020; Barboza da Silva et al., 2021a). In the peanut production chain, the insertion of digital technologies adapted for autonomous analysis of fungi can greatly enhance the sanitary rigor of seeds produced around the world (USDA, 2022). In addition, it indirectly reduces the uncertainties of contamination. These digital technologies have been explored by various imaging methods (Janik et al., 2021; Ziyaee et al., 2021). Therefore, by assisting our own abilities with artificial cognitive capabilities we can enhance our decision-making potential, and then use this for a purpose of global utility: high sanitary quality.

## Conclusion

We found multispectral markers of peanut seed sanitary states. Texture (Run Percentage), brightness (CIELab L*) and reflectance (490 nm), analyzed together, are highly effective to discriminate pathogenic fungi (*A. flavus*, *A. niger*, *Penicillium* sp. and *Rhizopus* sp.). The incubation period of 216 h for the multispectral analysis of the fungi allows discriminating them precisely in peanut seeds. The autonomous analysis of these markers is an opportunity to advance the accuracy of peanut sanitary determination in the seed industry. Thus, multispectral images coupled with machine learning algorithms are effective (from 90 to 100%) for screening peanut seeds with superior sanitary quality.

## Data availability statement

The original contributions presented in the study are included in the article/**Supplementary Material**. Further inquiries can be directed to the corresponding author.

## Author contributions

CM generated the research ideas. CM and JS collected multispectral image analysis data. GF wrote and formatted the manuscript. AM analyzed the data. CM, GF, TM, VA, and EA reviewed the manuscript, rewriting, discussing, and commenting. All authors contributed to the article and approved the submitted version.
