# Supplementary material for: Fungal identification in peanuts seeds through multispectral images: Technological advances to enhance sanitary quality
Source: Front Plant Sci. 2023 Feb 22;14:1112916. doi: 10.3389/fpls.2023.1112916 (PMC9992408; doi:10.3389/fpls.2023.1112916)
Supplement: Supplementary file 1 [file DataSheet_1.docx]

**Supplementary Material**

**Table S1 –** Performance metrics of machine learning models based on linear discriminant analysis – LDA, multi-layer perceptron neural network – MLP, random forest – RF, and support vector machine – SVM using a test dataset for classifying healthy peanut kernels and kernels contaminated with *Aspergillus flavus*, *Aspergillus niger*, *Penicillium* sp. and *Rhizopus* sp. using texture, color and reflectance descriptors.

| Metrics | Texture | | | |
| --- | --- | --- | --- | --- |
|  | LDA | MLP | RF | SVM |
| Accuracy | 0.54 | 0.67 | 0.58 | 0.66 |
| Kappa | 0.42 | 0.59 | 0.47 | 0.57 |
| Precision | 0.54 | 0.66 | 0.59 | 0.66 |
| Recall | 0.53 | 0.66 | 0.57 | 0.65 |
| F1 | 0.53 | 0.66 | 0.57 | 0.65 |
|  | Color | | | |
| Accuracy | 0.54 | 0.72 | 0.78 | 0.82 |
| Kappa | 0.42 | 0.64 | 0.73 | 0.78 |
| Precision | 0.55 | 0.73 | 0.79 | 0.82 |
| Recall | 0.53 | 0.71 | 0.78 | 0.82 |
| F1 | 0.53 | 0.71 | 0.78 | 0.82 |
|  | Reflectance | | | |
| Accuracy | 0.97 | 0.94 | 0.98 | 0.80 |
| Kappa | 0.97 | 0.92 | 0.86 | 0.75 |
| Precision | 0.98 | 0.94 | 0.89 | 0.87 |
| Recall | 0.97 | 0.94 | 0.88 | 0.79 |
| F1 | 0.97 | 0.94 | 0.88 | 0.79 |

**
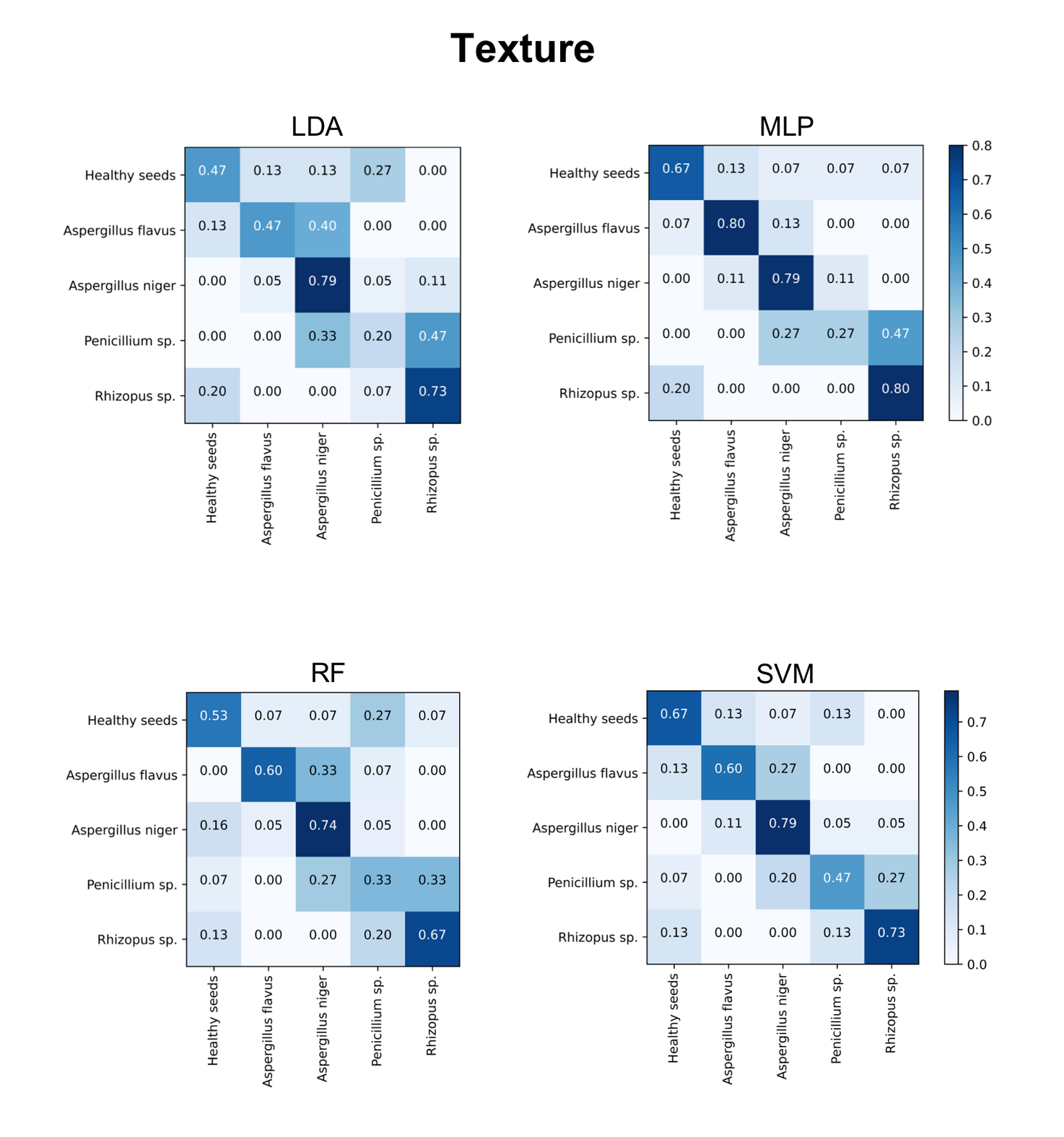
** **Supplemental Fig. S1.** Confusion matrices of machine learning models based on linear discriminant analysis – LDA (A), multi-layer perceptron neural network – MLP (B), random forest – RF (C) and support vector machine – SVM (D) using a test dataset for classifying healthy peanut kernels and kernels contaminated with different groups of fungi using texture descriptors.


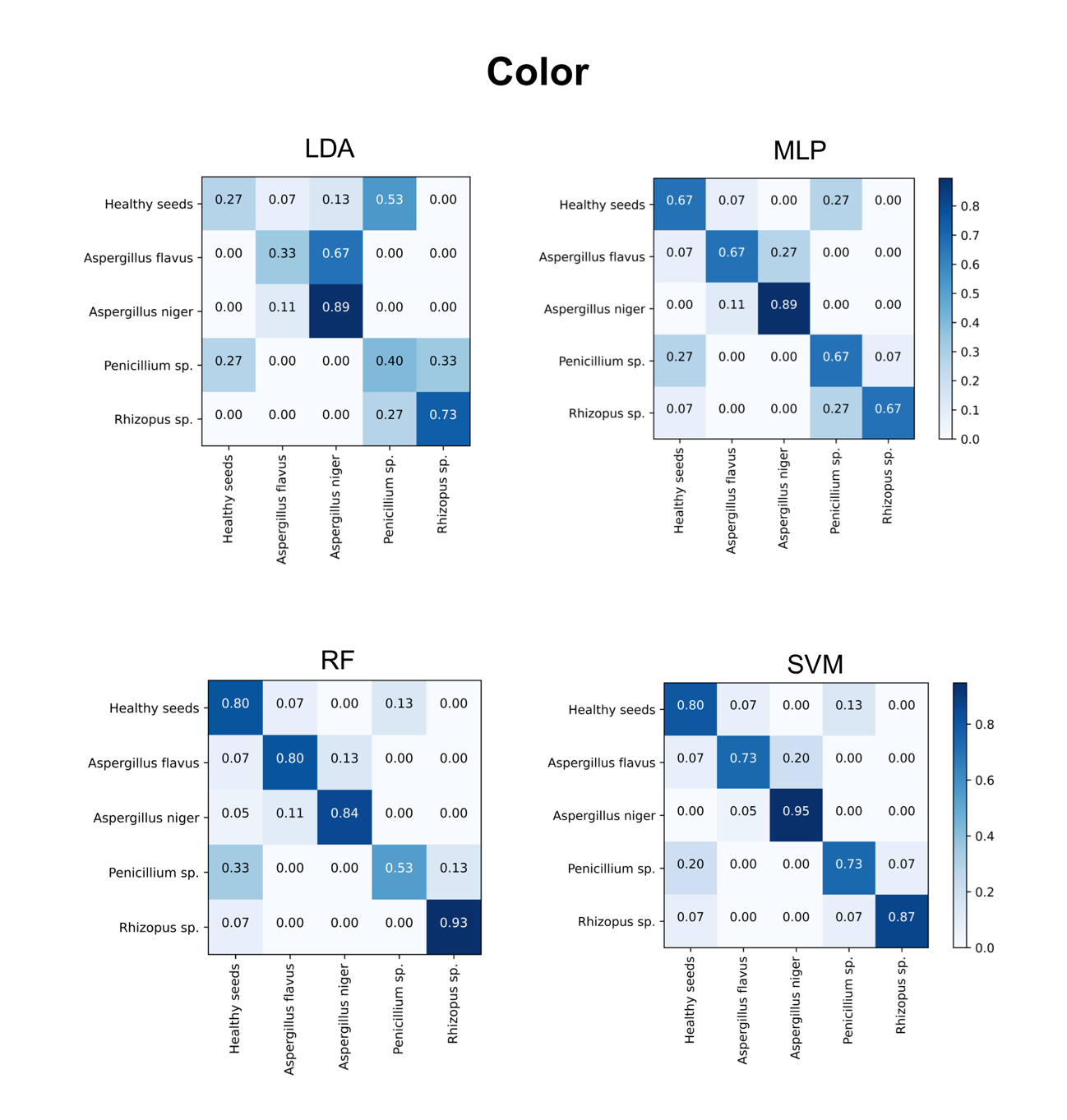


**Supplemental Fig. S2.** Confusion matrices of machine learning models based on linear discriminant analysis – LDA (A), multi-layer perceptron neural network – MLP (B), random forest – RF (C) and support vector machine – SVM (D) using a test dataset for classifying healthy peanut kernels and kernels contaminated with different groups of fungi using color descriptors.

**
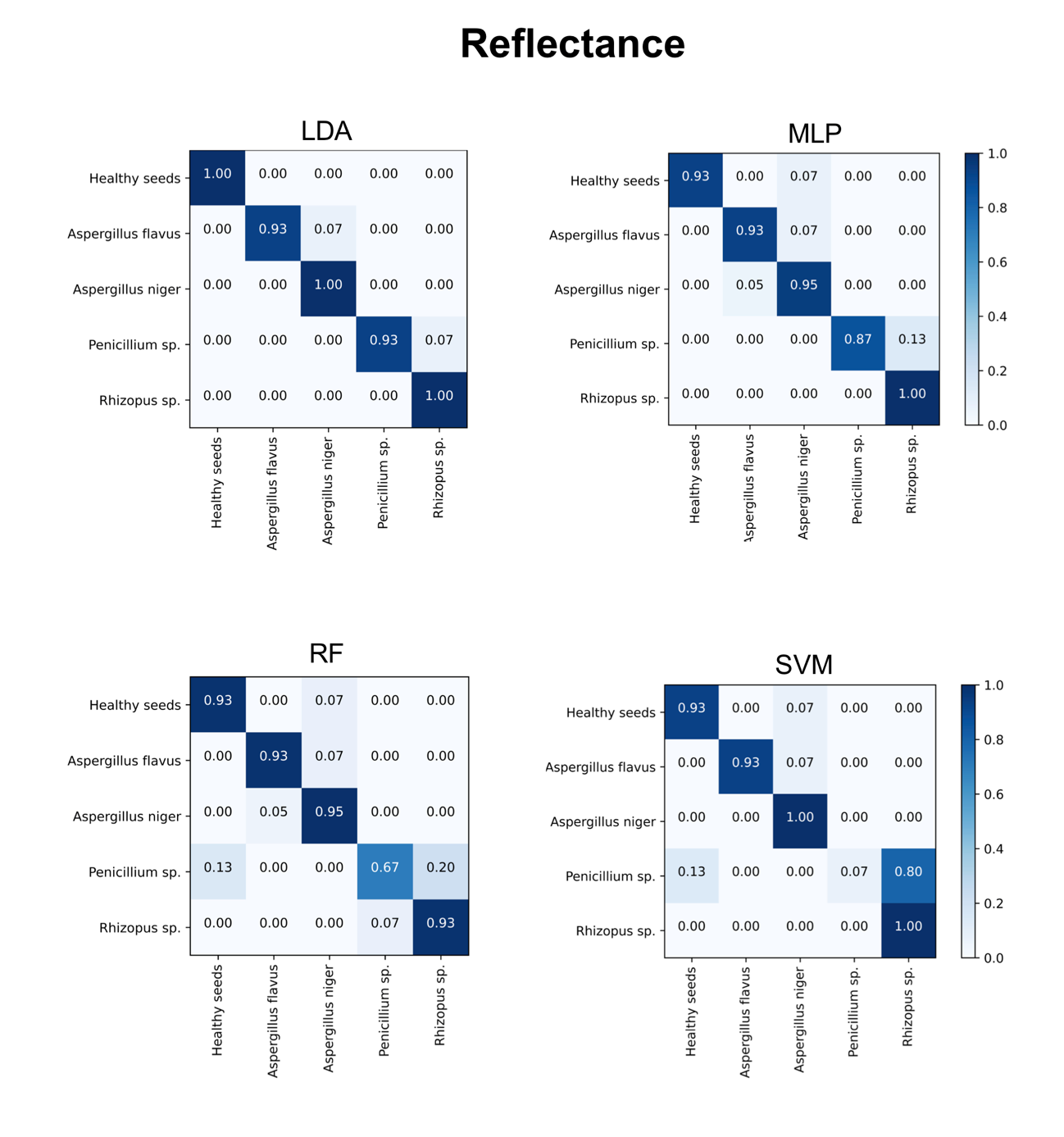
**

**Supplemental Fig. S3.** Confusion matrices of machine learning models based on linear discriminant analysis – LDA (A), multi-layer perceptron neural network – MLP (B), random forest – RF (C) and support vector machine – SVM (D) using a test dataset for classifying healthy peanut kernels and kernels contaminated with different groups of fungi using reflectance descriptors.
